# Innovative Training with Virtual Patients in Transcultural Psychiatry: The Impact on Resident Psychiatrists’ Confidence

**DOI:** 10.1371/journal.pone.0119754

**Published:** 2015-03-20

**Authors:** Ioannis Pantziaras, Uno Fors, Solvig Ekblad

**Affiliations:** 1 Cultural Medicine Unit, Department of Learning, Informatics, Management and Ethics (LIME), Karolinska Institutet, Stockholm, Sweden; 2 Department of Computer and Systems Sciences, Stockholm University, Stockholm, Sweden; University of Perugia, ITALY

## Abstract

**Background:**

Virtual patients are now widely accepted as efficient and safe training tools in medical education, but very little is known about their implementation in psychiatry, especially in transcultural clinical care of traumatized refugee patients.

**Objective:**

This study aimed at assessing the impact of training with a virtual patient on confidence in providing clinical care for traumatized refugee patients.

**Methods:**

The authors developed an educational tool based on virtual patient methodology portraying the case of “Mrs. K”, a traumatized refugee woman with symptoms of PTSD and depression. A group (N=32) of resident psychiatrists tested the system and their confidence in different aspects of providing clinical care for this patient group was evaluated pre- and post-test by using a validated confidence questionnaire. Cronbach’s α was calculated for all clusters. Changes between pre- and post-test were compared by using the matched-pair t-test, binomial distribution for exact significance test and a calculation of effect sizes (Cohen’s d).

**Results:**

A statistically significant improvement was exhibited in overall confidence (mean Δ: 0.34; p<0.0001; d: 0.89) as well as in four more specific domains of clinical care, with the area of identifying and evaluating trauma-related diagnoses and disability showing the most prominent improvement (mean Δ: 0.47; p<0.0001; d: 1.00).

**Conclusions:**

This VP-system can lead to physicians’ improvement of confidence in providing transcultural clinical care for traumatized refugee patients. Further research is required to investigate improvement in actual performance and cognitive outcomes with several VPs and in a long-term effect perspective.

## Introduction

Virtual Patients (VPs) have been technologically developed and researched since the 1990s as educational tools used to train different aspects of medical care, most commonly clinical reasoning, bioethics, clinical decision-making, leadership, and history taking [1–4]. They are broadly defined as “interactive computer simulations of real-life clinical scenarios for the purpose of medical training, education, or assessment” [5] and result in many advantages, some of them being easy accessibility, exposure to critical yet rare cases, training of skills in a safe environment, and standardization [6]. VPs have previously demonstrated an equivalent efficiency in improving the clinical performance and diagnostic ability when compared to standardized patients [7] but found to provoke lower engagement and interest levels compared to real humans [8].

While VPs have been extensively used in various areas of medicine integrating in some cases exciting technological capabilities like natural language dialogue and emotion reasoning [9], they are a rather new paradigm of education in the field of psychiatry [10]. A few studies have been published that involved the use of VPs for training assessment of suicide risk in general [11] and in bipolar patients [12], as well as diagnosing conduct disorder [13, 14], posttraumatic stress disorder in a teenager patient (PTSD) [15], schizophrenia [16], depression with panic disorder [17] as well as major depressive episode presenting with the chief complaint of fatigue and anhedonia [18]. Web-based simulation has been recently reported as a mean to teach violent risk assessment of complex psychiatric patients and the clinical management of follow-up care [19]. To the best of our knowledge, no previous study has been published concerning the formal use of VPs for transcultural training clinical management of traumatized refugee patients, with the exception of previous pilot studies published by our research team examining educational aspects of a preliminary version of our VP-system [20, 21] as well as the learners’ expectations and attitudes towards the current version [22].

We consider transcultural psychiatry to be a crucial domain of systematized knowledge, since the rapid increase of globalization (including migration and especially refugees) leading to more culturally diverse societies [23] has given rise to better understanding of the serious and often long-lasting consequences of the processes of migration and asylum seeking, reception, and exposure to traumatic life events before, during and after arrival in a receiving country. Severe mental disorders such as PTSD, depression [24, 25], cardiovascular disease [26], metabolic syndrome [27], as well as social disability as reflected by unemployment following hospitalization due to mental disorder [28], have been found to have a higher relative risk among immigrants, especially refugees, than in host populations.

We have developed a dedicated VP system that portrays refugee trauma cases for educational use, aiming to enhance the clinical, interpersonal, and cultural competence of trainees, as well as the clinical management of traumatized refugee patients. This system, including a slightly modified version developed as a part of our international cooperation with the Harvard Program for Refugee Trauma (HPRT) [20], has been evaluated with respect to user acceptance and educational potentials, as well as face and construct validity, with promising results [21, 22]. This study aimed to examine the impact of training with this VP-system on confidence in caring for traumatized refugee patients among Swedish psychiatry residents.

## Method

### Study Subjects

Thirty-two residents in psychiatry at the Karolinska University Hospital, Stockholm and University Hospital, Linköping, Sweden, volunteered to participate in our study in response to an e-mail invitation sent by the respective department director of studies from each hospital after communication with the research team. The director of studies for resident doctors in Sweden has as main responsibility to plan the overall study program of resident doctors, ensure the quality of it and offer advice and support during the training period to the residents as well as to their supervisors [29]. All of the residents that were active and not engaged by other clinical or educational duties at the time, accepted to participate. Twenty out of 32 participants (62.5%) were women and 12 (37.5%) were men. Their mean age was 35.6 years (female: 35.5, men: 35.9; range: 28–51) while mean time of working experience as psychiatry residents was 2.3 years (female: 2.4, men: 2.1; range: 0–5). No statistically significant differences in age and work experience were observed between male and female participants.

The Regional Ethical Committee at the Karolinska Institutet examined this study and its protocol and determined that because the project does not include any sensitive personal data and it is not considered research according to the ethics law, formal ethical approval was not required. The authors had access to information about age, sex, email address and work experience in years. This information falls under the Swedish definition of a research register bank according to the Personal Data Law. Because of that a separate application was sent and approved by the legal counsel at University Administration at the Karolinska Institutet. Data was anonymised prior to analysis.

### Materials

**Fig 1:**
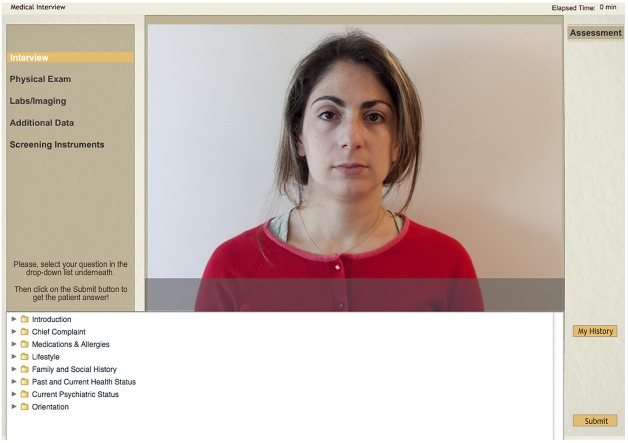
Screenshot of the Virtual Patient system illustrating the history-taking interview. The user can interact with the patient by choosing relevant questions divided in searchable appropriate categories and sub-categories. For illustrative purposes only, used with written permission by the portrayed model.

By using Adobe Flash CS4 Professional we developed a VP system called RT-Sim (Refugee Trauma Simulation), intended to train how to manage refugee patients. The current VP case tested in this study portrays a 45-year-old female Bosnian refugee patient (“Mrs. K”) with previous exposure to severe trauma, presenting with serious symptoms of PTSD, major depression and headache (Fig. 1). The VP is presented as a series of pre-recorded video clips that are displayed according to the questions asked by the user. Generic video sequences (i.e., the patient waiting for the doctor to ask a question, coughing, crying, etc.) were also utilized in order to achieve a higher degree of realism. During the interaction with the VP, the user was able to perform a medical interview and physical examination, use screening instruments and order selected laboratory and imaging tests. The medical interview was conducted by selecting appropriate questions from a list of questions (n = 148) divided suitably into different categories (i.e., reason for contact, past medical history, family history, social situation and current problems). Upon completion of the consultation with the VP, the user was asked to provide a preliminary assessment including a brief summary of the patient’s history, social situation and current symptomatology, as well as a preliminary diagnosis and a proposed treatment plan. This was followed by a detailed, individualized and automated feedback module delivered by: (a) the VP herself giving the patient’s perspective on different aspects of the consultation, and (b) the Virtual Advisor (VA) (“the expert”), giving detailed delayed feedback regarding clinical aspects of the consultation and basic communication skills. The provided feedback was structured and formed based on pre-determined and programmed rules, depending on the learners’ actual performance during the virtual interaction and included short and relevant theoretical background. For example, if the learner did not sufficiently examine the patient’s symptoms regarding intrusive recollection of traumatic events, the VA commented:

“You didn’t obtain sufficient information about experiences of intrusive recollection of the patient´s traumatic event like for instance images, thoughts, or perceptions, recurrent distressing dreams of the event, acting or feeling as if the traumatic event were recurring. Even physiologic reactivity upon exposure to internal or external cues that symbolize or resemble an aspect of the traumatic event can be experienced.”

A comprehensive description of the RT-Sim system and the current VP case used, along with the participants’ acceptance, expectations and attitudes towards it have been presented in previous articles [20–22].

### Outcome Measures

Confidence addressing mental health problems after trauma: In order to investigate the participants’ confidence in providing medical care for traumatized refugee patients before and after the training session with the VP, we used an instrument developed by the Harvard Program for Refugee Trauma (HPRT) that examines confidence in different areas of health and illness, communication with the patient, effects of diet and exercise on health, as well as different aspects of conducting related research and teaching activities. This validated, self-administered instrument, which we refer to as the “HPRT Confidence Questionnaire,” consists of 64 six-point Likert items and takes approximately 30 minutes to complete. It has been extensively used by HPRT for evaluating the effects of training programs for local health providers in countries like Cambodia [30]. All items measure the physician’s confidence in performing certain medical procedures by asking: “How confident are you that you can…” and scoring from 1 (= not at all confident) to 6 (= extremely confident). The participants were hence asked to predict their performance on a future task (predictive self-assessment).

For this study we revised the HPRT confidence questionnaire by excluding 20 items that were considered to not be relevant to the objectives of this study, in other words, those that examined aspects of planning and conducting research and educational activities as well as those that concerned non-relevant patient populations (children and adolescents).

### Procedure

All data was collected during 3 occasions (at each of the university hospitals), in quiet group rooms, where the participants had access to stationary or laptop computers and wired headphones. Upon signing the informed consent form, all participants were asked to complete a pre-test version of the HPRT confidence questionnaire. They were then given access to the RT-Sim system and were instructed to interact with the VP for up to 45 minutes and try to assess as they would do in a real-life clinical encounter. After interacting with the VP and receiving the provided feedback, the participants were asked to fill in the post-test version of the aforementioned questionnaire.

## Results

All of the participants (n = 32) completed the study and returned completed pre- and post-test questionnaires. The items of the HPRT confidence questionnaire (n = 44) were classified into 4 clusters that were comprised of similar items depicting 4 different aspects of medical care for traumatized refugee patients. Cronbach’s α was calculated for each cluster as well as for the whole HPRT confidence questionnaire in order to examine the internal consistency for the questionnaire and each cluster distinctly. Cronbach’s α > 0.7 was regarded as evidence for good internal consistency [31].

Table 1 presents the four generated clusters with examples of items, number of items included, and the estimated Cronbach’s α. The calculated Cronbach’s α for the overall questionnaire was 0.97 (44 items). The four clusters exhibited high values of Cronbach’s α as well (min: 0.87; max: 0.92), providing evidence for high internal consistency [32].

**Table 1:** The four generated clusters of confidence with examples of items, number of included items (n) and Cronbach’s α.

Cluster	Examples of included items	Items (N)	Cronbach-α
Identify and evaluate trauma-related diagnoses and disability	Identify post-traumatic stress disorder (PTSD); Ask about the patient’s/client’s "trauma story"; Use screening instruments	12	0.90
Treat and manage trauma-related diagnoses and disability	Treat trauma-related disability; Reinforce and teach positive coping behaviors for patients; Effectively use psycho-therapeutic medications	16	0.92
Treat victims of torture	Treat the mental health problems of torture survivors; Care for the spiritual problems of torture survivors; Care for the legal problems of torture survivors	6	0.88
Elements of intercultural communication	Adapt your work to different cultures and societies; Work effectively with an interpreter; Be culturally attuned to differences in meaning and interpretation of emotional upset between cultures	5	0.87

Table 2 demonstrates the confidence of the participants in different aspects of caring for traumatized patients, as self-reported before and after training with the VP and receiving the individualized feedback by the VP and the VA. The matched-pair t-test was used to estimate changes between pre- and post-test values of self-reported confidence. P-values ≤ 0.05 were considered as evidence of statistical significance. Effect sizes were calculated by using the equation: Cohen’s *d* = |M|/SD where *M* is the mean of differences (Δ), and *SD* is the standard deviation of differences. Effect size was interpreted according to the standard interpretation suggested by Cohen [21]: 0.8 = large (8/10 of a standard deviation unit); 0.5 = moderate (1/2 of a standard deviation); 0.2 = small (1/5 of a standard deviation).

**Table 2:** Changes in mean confidence scores between pre- and post-test, by cluster and gender.

N	Data	Mean	SD	Δ	SD	CI	d	t	p
**Overall questionnaire**									
All (N = 32)	Pre-test	3.86	0.73						
	Post-test	4.20	0.80	-0.34	0.38	-0.48, -0.21	0.89	-5.09	<0.0001
Male (N = 12)	Pre-test	4.13	0.21						
	Post-test	4.48	0.23	-0.35	0.46	-0.65, -0.06	0.76	-2.68	0.02
Female (N = 20)	Pre-test	3.70	0.70						
	Post-test	4.03	0.79	-0.34	0.34	-0.50, -0.18	1	-4.42	0.0003
**Identify and evaluate trauma-related diagnoses and disability**									
All (N = 32)	Pre-test	3.98	0.75						
	Post-test	4.46	0.83	-0.47	0.47	-0.64, -0.31	1	-5.73	<0.0001
Male (N = 12)	Pre-test	4.23	0.75						
	Post-test	4.65	0.88	-0.42	0.44	-0.69, -0.14	0.95	-3.31	0.007
Female (N = 20)	Pre-test	3.83	0.72						
	Post-test	4.34	0.80	-0.51	0.49	-0.74, -0.28	1	-4.61	0.0002
**Treat and manage trauma-related diagnoses and disability**									
All (N = 32)	Pre-test	3.92	0.81						
	Post-test	4.19	0.81	-0.27	0.33	-0.39, -0.15	0.82	-4.50	0.0001
Male (N = 12)	Pre-test	4.14	0.85						
	Post-test	4.45	0.80	-0.31	0.36	-0.54, -0.08	0.86	-2.99	0.01
Female (N = 20)	Pre-test	3.79	0.78						
	Post-test	4.03	0.79	-0.24	0.32	-0.39, -0.09	0.75	-3.30	0.004
**Treat victims of torture**									
All (N = 32)	Pre-test	3.29	0.92						
	Post-test	3.76	0.91	-0.46	0.69	-0.71, -0.22	0.67	-3.80	0.0006
Male (N = 12)	Pre-test	3.57	0.97						
	Post-test	4	0.86	-0.43	0.79	-0.93, 0.07	0.54	-1.88	NS
Female (N = 20)	Pre-test	3.13	0.87						
	Post-test	3.61	0.93	-0.48	0.63	-0.78, -0.18	0.76	-3.38	0.003
**Elements of intercultural communication**									
All (N = 32)	Pre-test	3.70	0.91						
	Post-test	4.02	0.98	-0.32	0.72	-0.58, -0.06	0.44	-2.50	0.02
Male (N = 12)	Pre-test	3.92	0.83						
	Post-test	4.35	0.86	-0.43	1.03	-1.09, 0.22	0.42	-1.46	NS
Female (N = 20)	Pre-test	3.57	0.95						
	Post-test	3.82	1.02	-0.25	0.47	-0.47, -0.03	0.53	-2.37	0.03

Pre-training confidence in all clusters was below 4 (“confident”), with male participants scoring higher than female (overall confidence in male participants = 4.13; female participants = 3.70), though not statistically significant (mean Δ = 0.43; CI: -0.10, 0.96; p = 0.11). Participants with working experience as residents in psychiatry over 2.5 years (n = 15) scored higher overall at baseline (mean: 4.03 ± 0.62) than those (n = 17) with less experience than 2.5 years (mean: 3.71 ± 0.81), a difference however, that was not statistically significant (mean Δ: -0.32; CI: -0.85, 0.20; p = 0.2).

There were statistically significant improvements in overall confidence after using the RT-Sim system (mean Δ: 0.34; p<0.0001; d: 0.89), as well as in all four clusters depicting more specific aspects of caring for traumatized patients. Effect size, measured by Cohen’s d, was overall large (0.89). Concerning 2 out of 4 clusters, the effect size was also large (> 0.8), whereas for the remaining two it was moderate (0.67) and small (0.44). The highest improvement was noticed in confidence in identifying and evaluating trauma-related diagnoses and disability (mean Δ: 0.47; p<0.0001; d: 1.00), in addition to confidence in treating and managing victims of torture (mean Δ: 0.46; p = 0.0006; d: 0.67). Whereas there was overall statistically significant improvement in all clusters, when analyzing by gender, it was found that confidence among male participants regarding (a) aspects of intercultural communication and (b) treatment of torture victims, was improved, but not to a statistically significant degree.

## Discussion

A previous pilot study by our team examined a preliminary version of the VP system presented in this study in terms of user acceptance and educational potentials [21], whereas a recently published study [22] examined the new version of our VP system with respect to users’ expectations and attitudes toward it. In this current study we examined the actual impact of training with VPs on the resident psychiatrists’ confidence in providing medical care for traumatized refugee patients with psychiatric symptomatology. It is, to the best of our knowledge, the first published study to examine the impact of VP on confidence in providing psychiatric care for a patient group in a broader context, while one previous pilot study examined the impact of VPs on confidence within a very specific slice of a clinical encounter (completing an informed consent task) [33].

Our current results revealed a statistically significant improvement and large effect size in overall confidence, as well as statistically significant improvement in confidence in specific domains of medical care for traumatized refugee patients, as defined by the four clusters used: (a) To identify and evaluate trauma-related diagnoses and disability (large effect size), (b) to treat and manage patients with trauma-related diagnoses and disability (large effect size), (c) to treat and manage victims of torture with mental illness (moderate effect size) and (d) effectively use elements of intercultural communication in the clinical praxis (small effect size). The highest improvement in confidence was observed in the domains of identifying and evaluating trauma-related diagnoses and disability, as well as in treating and managing victims of torture. The overall questionnaire (HPRT confidence questionnaire) as well as the generated clusters demonstrated very high Cronbach’s alpha, which speaks for more than acceptable internal consistency and high reliability.

This version of VP system has been previously evaluated in terms of user’s acceptance, expectations and attitudes with very promising results. The provided delayed summative, automated and individualized feedback was previously reported as very important elements of the program and valuable sources of knowledge. An innovative feature of this system was the provided feedback by the VP herself, giving needful and appreciated insights on aspects of medical care as seen from the patient’s viewpoint. Moreover, the design of the VP system favors a dynamic interaction between the user and the VP, as the VP’s reactions and answers depend partly on the user’s choices, questions asked and accomplished trust building. This allows the user to actively train in medical history taking, physical examination, ordering laboratory tests and planning treatment for this vulnerable patient group [6]. An important limitation of the system is however the lack of the ability to formulate in written or oral form own questions during the history-taking interview, limiting the perceived realism, as demonstrated in previously published studies. [21, 22]

It must be stressed that this study demonstrated the self-reported improvement in the participants’ confidence in providing medical care for refugee-traumatized patients and not any actual change in clinical performance or competence. This should be considered as a limitation of our study, since there are currently indications that physicians have a limited ability to accurately self-assess their competence [34]. However, there are also several studies that demonstrated positive association between self-assessed competence and external observations which give support to the theory that for at least some aspects of care, self-belief in one’s ability to perform a medical task may approximate one’s competence [35]. Fernandez et al. [36] demonstrated a strong association between self-rated language and cultural competence and patient satisfaction using an established interpersonal proof of care instrument. Similarly, Robbins et al. [37] demonstrated an association between self-reported “sensitivity to emotional and psychological issues in patients,” and the actual diagnosis of these issues in practice as audited by a blinded reviewer. Further research in the field of the relationship between self-reported confidence and actual competence in clinical, interpersonal and cultural aspects of medical care is definitely required. Other limitations in this study include the possible introduction of selection bias due to the non-random inclusion of our participants.

Despite the above-mentioned limitations, we consider this study to deliver important insights about the possible positive outcomes of training with VPs in psychiatric residency programs and to bolster previous promising findings about the application of VPs in psychiatric education in general [11–22, 38]. We strongly believe that with further development and fine-tuning, VPs can also act—as even previously suggested by a published study [27]- as a useful complementary tool in the process of assessing different aspects of psychiatry residents' clinical competence. The next step in our project is to examine actual cognitive outcomes pre- and post- training with our VP-system. Future studies will include a randomized control study examining actual patient care outcomes.
